# A review of what is an emerging contaminant

**DOI:** 10.1186/1752-153X-8-15

**Published:** 2014-02-26

**Authors:** Sébastien Sauvé, Mélanie Desrosiers

**Affiliations:** 1 Department of Chemistry, Université de Montréal, PO Box 6128, Succursale Centre-ville, Montreal, QC H3C 3J7, Canada; 2 Centre d’expertise en analyse environnementale du Québec, ministère du Développement durable, de l’Environnement, de la Faune et des Parcs, 2700 Einstein Street, Quebec City, QC GIP 3W8, Canada

## Abstract

A review is presented of how one defines emerging contaminants and what can be included in that group of contaminants which is preferably termed “contaminants of emerging concern”. An historical perspective is given on the evolution of the issues surrounding emerging contaminants and how environmental scientists have tackled this issue. This begins with global lead contamination from the Romans two millennia ago, moves on to arsenic-based and DDT issues and more recently to pharmaceuticals, cyanotoxins, personal care products, nanoparticles, flame retardants, etc. Contaminants of emerging concern will remain a moving target as new chemical compounds are continuously being produced and science continuously improves its understanding of current and past contaminants.

## Review

Emerging contaminants have now become a fashionable and trendy research venue. The large number of emerging contaminants poses a challenge for regulatory agencies. How to prioritize research about emerging contaminants? How to prioritize the definition of quality criteria or norms for all of these new substances for which we generally have only sparse knowledge on their behaviour in the environment or on their toxic effects on human health or the environment? The vogue for emerging contaminants certainly partly arises from the need of academic researchers to raise interest in their work and help finance their research projects. Researchers need to adapt to current trends if they want to be successful in raising funds.

The “emergence” of the awareness of emerging contaminants should probably be attributed to Rachel Carson for her 1962 book “Silent Spring” [1]. She convincingly showed that the widespread usage of DDT to eliminate mosquitoes and other pests had led to the death and disappearance of many birds – hence the title of the book. Carson was heavily criticized at the time for daring to challenge all of the benefits to society that arose from using pesticides in general and more specifically DDT. History proved her right and DDT was later banned and this is a good example of how an environmentalist rang the alarm bell and then academic research followed up to back things up with factual data and uncover the truth and risks involved with DDT – which again was first synthesized about a hundred years before Carson’s book and began to be spread generously during the second World War. We owe her for the eye-opening message that pesticides and chemicals in general can be problematic.

Once we focus on “emerging contaminants”, we need to better define what is being targeted. Given that qualification of what is “emerging” is relative, what was emerging as an important environmental contamination issue a decade or two ago, might no longer be qualified as an emerging contaminant. Within a broader context, one could extend the focus on emerging contaminants (contaminants which have appeared only recently) from contaminants of emerging concerns (contaminants which have been in the environment for a while but for which concerns have been raised much more recently). Finally, we could also incorporate emerging issues about more traditional contaminants (new facts or information which shed a new perspective on the concerns of well-known villains). This is actually our focus, looking globally at: i) “true or really new” emerging contaminants, new compounds or molecules that were not previously known or that just recently appeared in the scientific literature, ii) contaminants of emerging interest which were known to exist but for which the environmental contamination issues were not fully realized or apprehended, and iii) we also wish to tackle emerging issues about “old” contaminants, i.e., situations where new information is jostling our understanding of environmental and human health risks related to such legacy contaminants.

**Figure 1:**
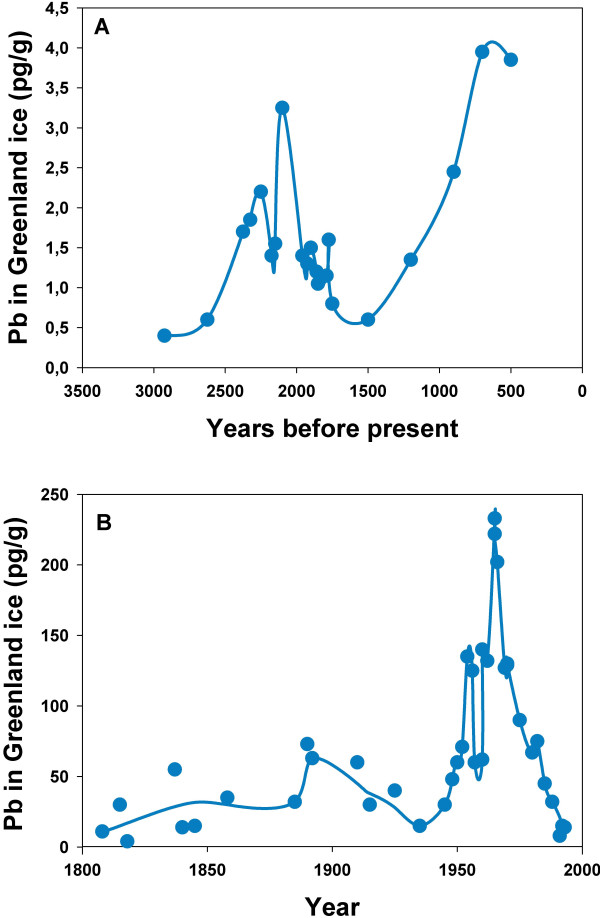
**Concentration of Pb in Greenland ice – redrawn from [**[2]**]. (A)** Data from 3000 to 500 years before present **(B)** Data for the last two centuries.

For an historical perspective on emerging contaminants, we would begin with what we believe might be the oldest global contaminant, lead. Its exploitation by Ancient Greeks and Romans was already on a scale large enough to be recorded in the polar ice banks of that era, see Figure 1[2]. When one looks at estimates of global lead production throughout the ages, one finds that the first steps of metal working and Pb production began about 5000 years ago. There was a noticeable increase around the apogee of Athens and a major increase in production arising from the exploitation of lead mines during the Classical era of the Roman Empire [3]. This production peak is only challenged about 2 millennia later, with the industrial revolution. One could argue that Pb was an emerging contaminant and the senators of Rome should have discussed the importance and potential toxicity of Pb that resulted from all the toxic metal leaching off luxuries of the time that depended on Pb-based metal containment. We could also bet that air, water and soil pollution must have been rampant around the mines of that era (worker/slave safety might not have been of high priority at the time). But even though Pb was emerging as a contaminant – there was no interest by those people because they had no clue of the risks Pb could cause and no means of measuring the trace (or not so trace!) levels of Pb in their environment. There might be some after the fact indications that Romans were indeed affected by Pb, but chances are that they were not aware of it.

Concerns that widespread contamination of our environment by Pb was detrimental to our health seemed to have become prevalent around the 70s. We could probably say that at the time, Pb was a contaminant of emerging interest – hard to say it was an emerging contaminant – that would have been 2 millennia too late! We would emphasize that when we use the term emerging contaminants – most of the time we really mean and focus on contaminants of emerging concern.

On the positive side, once we realized that Pb was detrimental to our health and our environment and strongly curtailed its use, we could observe a clear reduction of Pb concentration as recorded in Greenland snow records. In this case, widespread global contamination by Pb has been under control and current snow records seem comparable to conditions prevailing two centuries ago [4]. This illustrates that when we put a concerted effort, we can actually observe some significant global environmental improvement. We should nevertheless emphasize that comparing Figure 1A and B shows that cleaner Greenland ice levels are now still roughly twenty times above what they were before the humans’ first attempts at metallurgy.

**Figure 2:**
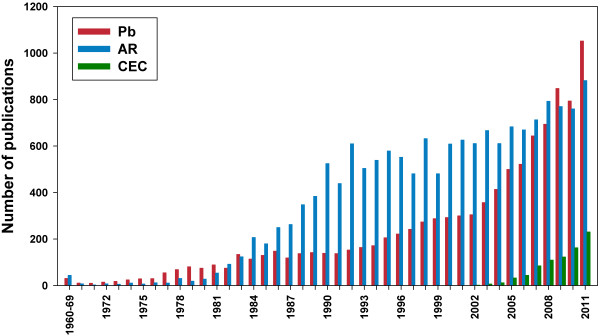
Number of yearly scientific articles on “Acid rain” or “Acid deposition” (AR), “Lead in drinking water” (Pb) or “Contaminants of emerging concern” (CEC), as can be found through a literature search in ScienceDirect.

This does not mean that we are done with Pb problems and that Pb will no longer be on our radar. On the contrary – to illustrate this, we repeated the exercise of Hua et al. [5] and compiled scientific articles published in ScienceDirect that included “Pb in drinking water” – (literature searches based solely on “lead” or “Pb” are not useful because they lead to tons of hits from the verb “to lead” and various derivatives and also numerous papers on Pb having nothing to do with contamination issues). Figure 2 shows that scientific articles dealing with Pb in drinking water began around the mid ’70 followed by two plateaus in the ’80 and ’90 and have been continuously increasing in the last decade. Part of the interest is that epidemiologic work has improved in finesse and significant detrimental effects are observed at increasingly lower levels – thus pressing the need for more information on effects, links between soil and water pollution and adverse health effects (mainly epidemiology on children neuro-cognitive development in the case of Pb). The latest findings with drinking water also point out that we might have overly focused on dissolved Pb and that Pb particulates arising from water distribution systems still represent a significant route of toxic exposure [6] – another *emerging* issue for an old contaminant.

Another legacy contaminant, yet, of emerging interest is arsenic. Arsenic is a romantic poison, omnipresent in novels and throughout history. Apart from its criminal links with intentional poisoning, it seems that arsenic sulfides were already used as pesticides in China as early as 900 A.D. [7]. The main products that were later used in agriculture seemed to be Paris Green as- copper acetoarsenite (CH_3_COO)_2_Cu3Cu(AsO_2_)_2_ and lead arsenate as Pb_5_OH(AsO_4_)_3_ or PbHAsO_4_. Lead arsenate and eventually other arsenic formulations were then internationally adopted as pesticides or biocides because of their efficiency (toxicity to pests) and relative low phytotoxicity [7]. It was when DDT became widely available that arsenical pesticides became less prevalent and eventually banned when the possibility of crop transfer of arsenic was better understood. We must emphasize that at the time we banned the evil arsenic for the presumed less harmful DDT which later followed in the same steps.

In a similar fashion, we are currently phasing out organophosphate pesticides and replacing them with supposedly less harmful products such as glyphosate, pyrethrinoids and neonicotinoids. Also, somewhat in a reverse trends, after many legislations prohibited the use of the highly toxic tributyltins antifoulant biocide [8], novel antifouling agents are appearing such as zinc pyrithione [9] or arsenic oxides, acetoarsenite and sublimated arsenic polysulfides which are now used as biocides in the production of antifouling coatings [10].

Nowadays, arsenic remains a contaminant of emerging interest, in this case mostly because of major problems of underground water contamination in South-East Asia where changes in land use have modified underground aquifer water flows [11]. Changes in aquifer usage are thus now contributing to a major mobilization of geochemical arsenic. In this case the source of the As is not a standard pollution where industrial wastes or agricultural releases would have contaminated the environment, the man-made changes to the local hydrography are simply helping to dissolve As which is naturally present in the underground aquifers – and thus exacerbating its release in the water that is being pumped by users.

Environmental quality criteria are intimately linked to emerging contaminants. As a new compound begins to cause concerns; data accumulate on its environmental chemistry, ecotoxicological and human toxicity, as well as its epidemiology. This eventually results in government action to establish environmental guidelines or criteria to ensure adequate protection. In a similar sequence, compounds that are already regulated are often re-evaluated with the addition of new data. A classical example for this might be the successive lowering of the target for safe lead level exposure in children now targeting blood lead below 5 μg Pb/dl from the previously used threshold of 10 μg Pb/dl [12]. In the 1960s and 1970s, the accepted threshold for adverse effects in children was 60 μg Pb/dl [13]. This clearly shows that as the weight-of-evidence builds up, the accepted threshold and criteria are re-evaluated and for the most part tightened. Another example is water quality guidelines for As which used to be around 50–25 and moved to 10 μg L^-1^, with Canada further contemplating reducing it to 5 μg L^-1^[14]. Current drinking water criteria for As are around 10 μg L^-1^, but it must be emphasized that, as in many cases, this threshold is a compromise resulting from a cost-benefit analysis of the gains in reduced cancer risks and the cost associated with treating drinking water to further reduce the concentration of As [15,16]. Some studies suggest that 3 μg L^-1^ seems a more appropriate target to prevent cancer risks in excess of 10^-4^[17]. A drinking water quality criteria based on the technological feasibility would be set lower at 3 μg As L^-1^[16] but would stigmatize some areas with high As geochemical backgrounds and force the treatment of many underground drinking water sources. However, the estimated cancer risk resulting from current exposure to As is around 1 in 10?000 instead of the normally accepted threshold of 1 chance in a million [15,18,19]. Consequently, we can suspect that further epidemiological work might redefine the acceptable threshold for cancer risks related to arsenic exposure and that the drinking water quality criteria for As are prone to be eventually revisited to integrate current and future knowledge on the problems caused by arsenic.

Lead and arsenic are old contaminants with different emerging concerns through human history. Other emerging issues have appeared more recently. The first example was an emerging issue in the ‘80s, “acid rain” or “acid deposition”. In this case we could observe a steady increase in scientific publications during the ’80s and a more or less steady flow of papers since the early ’90s (see Figure 2). In the last decade, we also observed new emerging issues related to chemical contaminants such as, sensitivity to toxic chemicals related to climate change, natural and anthropogenic chemicals related to hydraulic fracturing and shale gas exploitation, increase of mining activities related to rare earths or radionuclides, treatment by-products produced during water treatment (e.g. [20,21]), etc. What are the next issues of concern and which new or old contaminants will be related to them? What is the next challenge for scientists and regulatory agencies?

More “true or really new” emerging contaminants would of course include many more types of contaminants such as pesticides, pharmaceuticals and personal care products, fragrances, plasticizers, hormones, flame retardants, nanoparticles, perfluoroalkyl compounds, chlorinated paraffins, siloxanes, algal toxins, various trace elements including rare earths and radionuclides, etc. It is only a few examples from a long list of potential emerging contaminants. The exercise of a literature search was also done for the term “emerging contaminants” and we observed a steady increase since the turn of the millennia (Figure 2) but this probably does not reflect so much the scientific efforts towards contaminants of emerging interest but rather the coining of the term “emerging contaminants”.

An interesting example of emerging chemicals are the flame retardants. The first to appear were the PCBs, an important group of contaminants that were dispersed in the environment due to a strong industrial use between 1929 and 1977 [22]. Despite the fact that their use was banned in North America in the late 70’s, environmental problems resulting from their presence are still relevant due to their persistence in the environment, their toxic properties and their bioamplification along trophic food webs even as far as in polar systems [23-25]. Polybrominated compounds have in many cases replaced polychlorinated compounds and have also begun to appear in Arctic and Antarctic environments [26,27], even in polar bears [28], and some of them are now regulated. Several voluntary initiatives to reduce the use of polybrominated diphenyl ethers (PBDEs) have been undertaken since 2001, mainly in Europe but also in North America. These initiatives aim to eliminate the PentaBDE and OctaBDE. In Canada, a regulation was first held in 2006 for these compounds. As implemented in the U.S., Canada also supports the virtual voluntary elimination of DecaBDE by 2013 [29]. Industries are producing new brominated flame retardants which are now being measured in the environment [30,31] and show bioaccumulation in various organisms [32-34]. The new flame retardants products represent contaminants of emerging concerns and the persistence of the older ones such as PCBs and PBDEs are highly persistent in the environment and still an environmental problem particularly in aquatic systems where sediments are recognized as a sink for this class of contaminant.

Recent decades have seen the emergence of neonicotinoids as a new generation of pesticides, presumed more efficient and hence applied in smaller quantities. One can challenge the concept that using smaller quantities of a much more toxic chemical is environmentally sound and foolproof. Neonicotinoids form a new class of pesticides produced in significant quantities (e.g. imidacloprid, acetamiprid, thiamethoxam, etc.). This class of pesticide is specific to the nicotinic acetylcholine receptor (nAChR) of insects [35] and successfully applied to control pests and their natural enemy [36]. This new group of pesticides has become a major suspect of the troubles of pollinators, particularly honey bees [37]. What are their persistence in the environment and their effect on other non-target insects, or on the aquatic larval stages of many species? Another regulatory challenge associated with the risk assessment of this CEC is currently debated within the EU who has enacted a two-year ban on their use to protect honey bees [38].

Pharmaceuticals have emerged in the past decade or two as emerging contaminants (albeit pharmaceutical drugs have been consumed for much longer than that), naturally occurring hormones are also often analysed and studied along with synthetic steroids and are strong endocrine disruptors. Natural steroid hormones must have been released into wastewaters and must have affected the rivers of early human settlers (albeit on a small scale). The emerging issue is that since we are continuously improving our ability to detect various emerging contaminants (e.g., [39,40]) we have realized that the presence of human or veterinary pharmaceuticals is rampant in surface waters [41]. Estrogenic hormone concentrations in wastewater effluents and receiving surface waters are often well above the recognized threshold for feminization of fish [42] and traces of various pharmaceuticals can even be found in drinking water. It is debatable whether we need to be concerned over the exceedingly low concentrations of standard pharmaceutical drugs that are usually observed in drinking water [43,44]. Nevertheless, traces of anti-infective pharmaceuticals in surface waters and urban effluents have also been shown to be more than sufficient to induce antibiotic resistance [45].

Plasticizers and their metabolites are measured in the environment [46] and in sewage treatment plant effluents [47]. Plasticizers, are additives used to increase the flexibility or plasticity, such as bisphenol A or phthalates and are particularly recognized as an endocrine disruptors [48] and have been under scrutiny with some of the plasticizers already having been banned or more strictly regulated.

Various fluorinated compounds, mainly perfluoroalkyls and polyfluoroalkyl (PPFAs) have made it to the market and have since has been targeted for stricter regulations given their environmental properties. For example, in Canada, regulations published in 2008 prohibit the manufacture, use, sale, and import of PFOS, its salts, and its precursors. Five-year exemptions provided for aqueous film forming foams and PFOS-based fume suppressants used in the metal plating industry expire in 2013. PFOs still represent an environmental problem because of their potential for long-range transport [49], high persistence in the environment, high bioaccumulation potential in organisms in polar environments [49,50] and it is suspected to show significant immunotoxic [51] and hepatotoxic toxicity [52].

A different type of contaminant is that of cyanotoxins, cyanobacteria are among the first biological organisms on Earth, so not really a newcomer. Nevertheless, eutrophication of water bodies and global warming are contributing to algal blooms and improved analytical techniques now allow us to better detect cyanotoxins produced by those organisms [53]. Acute toxicity is possible and can be fatal but research on chronic exposure to low levels and the negative impact the pro-inflammatory responses they could illicit has not yet been properly documented [54]. Not all cyanotoxins have yet even been identified and only a subset is being monitored. For example, the most commonly found cyanotoxins are the microcystins. There are about 80 known variants of microcystins and only a few are commonly monitored. No more than a dozen of variants are currently available as analytical standards so that many known microcystins are not even monitored in water bodies recognized for the occurrence of cyanobacterial blooms.

Two other major groups of contaminants are also emerging, manufactured nanoparticles and treatment by-products. Treatment by-products are generated when water treatment (drinking or wastewater) is generating new products from the reaction of the reagents with the components of the matrix or when reactions of the target contaminants are incomplete and some by-products are generated that may have some residual toxicity [20,55]. For example, chlorination can generate by-products such as haloacetic acids or trihalomethanes [56]. Treatment can focus on conceptually simpler biological processes such as constructed wetlands which can nevertheless be quite efficient [40] or more complex chemical treatment such as oxidation with chloration or permanganate [57]. Ozone is often proposed as an alternative or additional treatment that could potentially reduce or eliminate such by-products but ozone itself is so reactive that it is also a major means of producing a suite of by-products, examples are given for the identification of the transformation products of antidepressor drugs [20] or for a natural estrogen [21]. Given that ozone is becoming increasingly popular, we can expect more work to emerge on identifying by-products of incomplete ozone treatment and their quantification. Within the realm of water treatment, one must also emphasize that low input technology such as aerated lagoons or constructed wetlands [40] certainly deserve better recognition for their potential for low-cost removal of emerging contaminants.

The case of nanoparticle is quite challenging. In this case, the risk assessment paradigm itself needs to be re-evaluated. Nanoparticles are defined as having at least one dimension which is less than 100 nanometers. The nanoparticles can further be subdivided between carbon-based nanoparticles such as carbon nanotubes or fullerenes and metal-based nanoparticles such as metal oxides or quantum dots.

The challenge in assessing the environmental or human health risks of nanoparticles is partly on how to measure them: they cannot be filtered out through conventional means, the nanoparticles being smaller than the filter’s pores. Ultrafiltration is a potential option to segregate those particles from their matrix but not something that is easily realized. Carbon-based nanoparticles behave partly like heavy organic compounds and partly like small particulates. Hence the study must integrate both aspects. Metal-based nanoparticles must also be evaluated in terms of their toxicity to help differentiating what portion of the toxicity arises from metallic components of the nanoparticles being dissolved into the media and compare that to the portion of the toxicity that could be attributed to the nanoparticle itself. Once absorbed into an organism, is it easily eliminated or too large to be filtered out in that organism? In the environment, do they quickly degrade into their metallic components – and hence we would not require a change of paradigm, just a good identification of the new portion of the periodic table which is being exploited and dispersed. If they are stable, do they aggregate, do they stay as single-particle colloids? How will they behave in a sewage treatment plant (e.g., [58])?, will they transform if dispersed into soils (e.g., [59])? If they aggregate (and they often do), is the aggregate stable, can they later re-disperse? There are much research efforts into the field of the environmental fate and risks of nanoparticles, but probably more questions than answers for the moment.

## Conclusions

Emerging contaminants and emerging issues about soil, air and water contamination have been around without our knowledge for millennia and we can hope that as environmental chemistry, health and environmental toxicology improves, we can do a better job to prevent deleterious effects upon our environment and our health and reduce the number of situations where we wait until the damage is rampant before we enforce some form of regulation.

To conclude, if we want a firm definition of emerging contaminants, we would actually be tempted to prefer defining *contaminants of emerging concern (CEC)* as naturally occurring, manufactured or manmade chemicals or materials which have now been discovered or are suspected present in various environmental compartments and whose toxicity or persistence are likely to significantly alter the metabolism of a living being. Such potential CEC should remain “emerging” as long as there is a scarcity of information in the scientific literature or there are poorly documented issues about the associated potential problems they could cause. In general, we expect CECs to be chemicals that show some potential to pose risks to human health or the environment and which are not yet subjected to regulatory criteria or norms for the protection of human health or the environment. Not all CECs will actually prove to be evil and have some potential to cause tangible concerns; the focus is that the lack of pertinent environmental fate and ecotoxicological or toxicological data prevent the proper evaluation of associated risks. An already regulated presumed well-known contaminant could certainly regain “emerging” status as new scientific information becomes available and thus force regulatory agencies to re-evaluate their norms and guidelines.

The challenges in the years to come will be to better understand contaminants of emerging concern, their concentrations in the environment as well as their toxic effects on organisms in order to achieve better manage risks to human health and the environment. In this era of budgetary restrictions, reducing research funding is being targeted as a means of saving money [60] but this is a short-sighted perspective which could have costly consequences in terms of environmental impact, loss of biodiversity and ultimately, consequences on human health.

## Competing interests

The authors declare that they have no competing interests.

## Authors’ contributions

SS and MD performed the data gathering and wrote and approved the final manuscript.
